# Astragalus membranaceus extract attenuates ulcerative colitis by integrating multiomics and the PI3K/AKT signaling pathway

**DOI:** 10.3389/fphar.2025.1585748

**Published:** 2025-06-09

**Authors:** Jiaying Zhu, Chenhuan Shentu, Qingling Meng, Sen Fan, Yunjia Tang, Mingjiang Mao, Xiaofeng Yuan

**Affiliations:** ^1^ School of Life Sciences, Zhejiang Chinese Medical University, Hangzhou, China; ^2^ Future Health Laboratory, Innovation Center of Yangtze River Delta, Zhejiang University, Jiaxing, China

**Keywords:** ulcerative colitis, Astragalus membranaceus, microbial dysbiosis, metabolites, PI3K/Akt signaling pathway

## Abstract

**Introduction:**

Ulcerative colitis (UC) is a chronic, non-specific inflammatory bowel disease (IBD) that severely impairs the quality of life of affected individuals. *Astragalus membranaceus* (*Fisch.*) *Bge*. [Fabaceae; Astragali radix] (AM), a widely used Chinese medicine, has active ingredients that exhibit significant anti-inflammatory effects. This study aimed to investigate the role of AM extract in alleviating dextran sulfate sodium (DSS)-induced colitis in mice.

**Methods:**

A UC model was established, and AM extract was orally administered to evaluate its role in the treatment of colitis in mice. The effects of AM extract on colitis mice were subsequently investigated via 16S rDNA profiling, as well as short-chain fatty acids (SCFAs) and bile acids (BAs) sequencing. In addition, network pharmacology approaches and supplementary validation experiments were conducted to elucidate the underlying mechanisms of action.

**Results:**

AM extract notably alleviated UC-related symptoms, promoted the restoration of intestinal barrier integrity, and suppressed inflammation. Concurrently, it contributed to the rebalancing of the gut microbiota and normalization of both SCFAs and BAs metabolic processes. Mechanistic investigations integrating network pharmacology and experimental validation revealed that the beneficial effects of AM extract are likely mediated through the regulation of the PI3K/AKT signaling pathway.

**Discussion:**

Our study demonstrated that the AM extract effectively alleviated UC. The extract was shown to modulate microbial dysbiosis triggered by DSS and promote the generation of beneficial metabolites. Subsequent analyses confirmed that these effects are mediated through activation of the PI3K/AKT signaling pathway. Collectively, these findings offer novel insights into treatment strategies for UC.

## 1 Introduction

IBD represents a chronic inflammatory disease affecting the gastrointestinal tract, encompassing Crohn’s disease and UC. UC is associated with symptoms such as abdominal pain, diarrhea, rectal bleeding, and weight loss (Guan, 2019). Its pathogenesis is influenced by various factors, predominantly dysregulated immune responses and epithelial barrier dysfunction, leading to persistent gastrointestinal damage (Nakase et al., 2022). Furthermore, the progression of UC markedly increases the risk of colorectal cancer, severely impacting patient survival (Gros and Kaplan, 2023). The existing therapeutic options for UC are often accompanied by severe side effects, suboptimal efficacy, and high recurrence rates, necessitating the identification of novel treatment strategies and therapeutic agents.

Recently, the gut microbiota has emerged as a critical area of research because of its roles in maintaining mucosal barrier integrity, preventing pathogenic invasion, and modulating immune responses (Qiu et al., 2022). Similarly, the imbalance of the gut microbiota and its metabolites in UC can damage the intestinal barrier, thereby exacerbating systemic inflammation. These metabolites interact with host receptors to influence immune and inflammatory responses, suggesting potential therapeutic targets for UC (Cai et al., 2022). Among these metabolites, SCFAs, including acetic acid, propionic acid, and butyric acid, are pivotal in enhancing intestinal barrier function. SCFAs mediate cytokine production by regulating monocytes and macrophages, exerting potent anti-inflammatory effects (Deleu et al., 2021). BAs are involved in bacterial transformation, including deconjugation, dehydroxylation, and dehydration, resulting in secondary BAs that are crucial for immune regulation. These secondary BAs suppress proinflammatory cytokine expression in various immune cells, including monocytes, macrophages, and dendritic cells (Fu et al., 2023; Guo et al., 2020). Consequently, SCFAs and BAs have the potential as novel anti-inflammatory agents or therapeutic targets for UC management.

Here, emerging evidence highlights the pivotal role of the phosphatidylinositol 3-kinase (PI3K)/protein kinase B (AKT) signaling pathway in modulating oxidative stress and inflammation. Upon activation, PI3K catalyzes the phosphorylation of phosphatidylinositol 4,5-bisphosphate (PIP2) into phosphatidylinositol 3,4,5-trisphosphate (PIP3) at the cell membrane. PIP3 subsequently activates phosphoinositide-dependent kinase 1 (PDK1), which phosphorylates AKT, leading to an increase in phosphorylated AKT (p-AKT) (Zarneshan et al., 2020). Notably, inhibition of the PI3K/AKT pathway has been shown to enhance intestinal barrier integrity, restore oxidative balance, and mitigate UC symptoms (Chen et al., 2017; Asaad and Mostafa, 2024). Zou et al. (2022) reported that stimulation of the PI3K/Akt signaling pathway enhances the expression of tight junction proteins—such as ZO-1, occludin, and claudin-1—which contributes to the restoration of epithelial barrier function compromised by IL-1*β*. Thus, modulation of the PI3K/Akt signaling pathway may serve as a promising strategy for decreasing intestinal barrier permeability.

As a treasure of traditional Chinese medicine (TCM), AM has been revered as the “King of Herbs” and recorded in the classic Chinese medicine “Shengnong’s herba”. AM has a variety of therapeutic effects and is commonly used to treat conditions such as diarrhea, rectal bleeding, and related gastrointestinal ailments (Auyeung et al., 2016). Modern pharmacological studies have revealed that AM contains bioactive compounds such as saponins, polysaccharides, and flavonoids, which exhibit therapeutic effects on various diseases through various mechanisms (Dong et al., 2022). Currently, AM extract has demonstrated well-documented anti-inflammatory properties in the treatment of both breast and gastrointestinal cancers (Zhou et al., 2018; Hou et al., 2023). However, its potential efficacy in addressing UC-related damage remains largely unexplored. Therefore, this study aims to preliminarily assess the therapeutic potential of AM extract in colitis mice by integrating multiomics analyses, network pharmacology approaches, and a series of validation experiments from multiple perspectives, providing a reference for the clinical treatment of UC with AM.

## 2 Materials and methods

### 2.1 Materials

The AM used in this study was obtained from Chinese Herbal Pieces Co., Ltd., Zhejiang Chinese Medical University (Hangzhou, China), and authenticated by Professor Jiaqi Guan of Zhejiang Chinese Medical University. Standards (calycosin 7-O-glucoside and ononin) were sourced from Yuanye Biotechnology Co., Ltd. (Shanghai, China). DSS was acquired from Yeasen Biotechnology Co., Ltd. (Shanghai, China). ELISA kits for IL-1*β*, IL-6, and TNF-*α* were obtained from Youke Biotechnology Co., Ltd. (Hangzhou, China), while kits for LPS and D-lac were supplied by Mei-mian Industrial Co., Ltd. (Yancheng, China). ZO-1, Occludin, PI3K, p-PI3K, p-AKT, and NF-κB antibodies were acquired from Huaan Biotechnology Co., Ltd. (Hangzhou, China). Antibodies targeting Akt, and Claudin-1 were sourced from Servicebio Biotechnology Co., Ltd. (Wuhan, China), while Nrf2 antibody was sourced from Abways Biotechnology Co., Ltd. (Shanghai, China). Chromatographic grade of Acetonitrile and Methanol were provided by Meryer Biotechnology Co., Ltd. (Shanghai, China).

### 2.2 Methods

#### 2.2.1 Preparation of AM extract

Specifically, 20 g of AM sample was soaked in 200 mL of 80% ethanol for 30 min, followed by extraction via the condensation reflux method for 1 h, which was repeated twice. The resulting filtrates were combined and concentrated via a rotary evaporator to a final concentration of 0.5 g/mL and then stored for subsequent experiments.

#### 2.2.2 Analysis of AM extract components using UPLC/Q-TOF-MS

The active components of the AM extract were assessed via an ultra-performance liquid chromatography-quadrupole-time-of-flight mass spectrometry (UPLC/Q-TOF-MS) system. Chromatographic separation was performed with a CORTECS C18 column (100 × 2.1 mm, 1.6 μm) maintained at 35°C. The mobile phase consisted of 0.1% formic acid in water (A) and acetonitrile (B). Gradient elution was executed at a flow rate of 0.3 mL/min with a 2 μL injection volume. The gradient protocol was structured as follows: 95% A and 5% B from 0 to 2 min; a gradient from 95% to 0% A and 5%–100% B from 2 to 32 min; 100% B from 32 to 33 min; 95% A and 5% B at 33.5 min; and 95% A and 5% B from 33.5 to 35 min.

#### 2.2.3 Preparation of the test solution, standard solution, and HPLC conditions

Of AM extract, as prepared in section “2.2.1”, 1 mL was centrifuged at 120,000 r·min^–1^ for 12 min to obtain the supernatant. The resulting supernatant was subsequently filtered through a 0.45 μm membrane to remove impurities, and carefully transferred into a sample vial to prepare the test solution. Additionally, 1 mg each of Calycosin-7-O-glucoside and Ononin were accurately weighed and dissolved in 10 mL of methanol to yield standard solutions with a concentration of 0.1 mg/mL, respectively.

HPLC analysis of Calycosin-7-O-glucoside and Ononin was performed using a Waters 2,695 system, with chromatographic separation carried out on an Agilent ZORBAX SB-C18 column (250 × 4.6 mm, 5 µm particle size). The mobile phase consisted of ultrapure water (solvent A) and acetonitrile (solvent B), delivered at a constant flow rate of 1.0 mL/min. The gradient elution program was as follows: 10% B at 0 min, increasing to 25% B at 4 min, 50% B at 10 min, 70% B at 20 min, held at 40% B until 25 min, and returned to 10% B at 30 min. The injection volume for each sample was 10 μL, with detection carried out at 220 nm, and the column temperature maintained at 33°C throughout the analysis.

#### 2.2.4 Animal grouping and administration

Thirty-five healthy male C57 mice were acclimated for 1 week before being randomly allocated into five groups: the control group, DSS group, L_AM group (75 mg/kg AM extract), H_AM group (150 mg/kg AM extract), and Mesalazine group (100 mg/kg) (Zhu et al., 2023). All groups, except the control group, received 2.5% DSS in their drinking water continuously for 9 days to induce UC symptoms, while the control group was provided regular drinking water. Drug treatments were administered concurrently during the modeling period. The development of symptoms such as diarrhea, loose stool, or bloody stool is considered indicative of successful UC modeling. The study received approval from the Scientific Ethics and Safety Committee of Zhejiang Chinese Medical University (SCXK [Zhejiang] 2021–0012; Hangzhou, China).

#### 2.2.5 Disease activity index (DAI) score

The body weight of each mouse was recorded daily, with observations of body surface hair condition, mental state, and fecal consistency. The o-toluidine method was employed for the qualitative detection of fecal occult blood. The DAI score was calculated on the basis of changes in body weight, fecal consistency, and fecal occult blood results (Wu et al., 2021). DAI scores were calculated using the following formula:

DAI = (body weight loss fraction + fecal consistency fraction + fecal occult blood fraction)/3.

#### 2.2.6 Draw materials

On the 10th day, blood was drawn from the submaxillary vein of each mouse. After they had rested for 50 min, the samples were centrifuged, and the supernatants were collected and stored at −80°C for subsequent analyses. The colon was subsequently peeled off carefully, and the contents were removed into a cryovial. The samples were frozen in liquid nitrogen for 5 min and then transferred to −80°C for storage. In addition, the spleen and thymus were carefully removed and weighed to calculate the thymus and spleen indices. Concurrently, the colon was isolated to measure its length, and the sections were preserved in 4% paraformaldehyde for pathological staining, while the remaining colon tissue was stored at −80°C for further experiments.

#### 2.2.7 Pathological staining

About 1 cm of colon tissue was fixed in 4% paraformaldehyde for 2 days. The tissue was then dehydrated via a graded ethanol series, cleared with xylene, and embedded in paraffin at 56°C. Sections of 4 μm thickness were prepared, stained with hematoxylin-eosin (H&E) dye solutions, sealed with neutral gum, and examined microscopically to evaluate pathological changes in the colon.

#### 2.2.8 Enzyme-linked Immunosorbent Assay (ELISA)

A portion of colon tissue was placed in a 1.5 mL centrifuge tube, mixed with nine times its volume of normal saline (NS), and ground twice for 2 min each. The mixture was subsequently centrifuged at 12,000 rpm for 10 min, after which the supernatant was collected. The levels of IL-1*β*, IL-6, and TNF-*α* in the colon tissue were measured using ELISA kits according to the manufacturer’s instructions. Additionally, ELISA was performed to determine the levels of LPS, D-lac, IL-1*β*, IL-6, and TNF-*α* in the serum.

#### 2.2.9 16S rDNA sequencing

16S rDNA sequencing for the identification of metabolites was completed by Hangzhou Cosmos Wisdom Biotech Co., Ltd. (Hangzhou, China). Total DNA was extracted from the samples, and primers targeting conserved regions with sequencing adapters at the ends were designed for PCR amplification. The amplified products were subsequently purified, quantified, and homogenized to construct a sequencing library. Libraries meeting quality standards underwent high-throughput sequencing using the Illumina NovaSeq 6,000 platform. The raw image data files were processed via base calling to generate sequenced reads. These sequences were subjected to quality control and denoising processes, and amplicon sequence variants (ASVs) were classified. Species identification was conducted on the basis of sequence composition.

#### 2.2.10 Targeted SCFAs sequencing

The targeted SCFAs sequencing for the identification of metabolites was completed by Hangzhou Cosmos Wisdom Biotech Co., Ltd. (Hangzhou, China). A volume of 100 μL of plasma was obtained from each mouse, to which 200 μL of methanol was added to ensure complete protein precipitation. Following centrifugation, the supernatant was collected and evaporated to dryness. The resulting residue was reconstituted in 30 μL of 80% methanol solution, and 5 μL of an internal standard (IS) solution was subsequently introduced. For derivatization, 10 μL of derivatization reagent A (200 mM 3-NPH in 75% aqueous methanol) and 10 μL of derivatization reagent B (96 mM EDC-6% pyridine solution in methanol) were added. The reaction was conducted at 30°C for 30 min. After completion, the mixture was centrifuged, and the supernatant was collected for analysis. Chromatographic separation was performed using a Thermo Vanquish Flex UHPLC system equipped with a Waters BEH C18 column (2.1 mm × 100 mm). The negative ion mode was used for detection, and quantitative data analysis was carried out with Xcalibur.

#### 2.2.11 Targeted BAs sequencing

The targeted BAs sequencing for the identification of metabolites was completed by Hangzhou KaiTai Biotech Co., Ltd. (Hangzhou, China). An aliquot of 100 μL of the plasma sample was accurately measured, followed by the addition of 500 μL of precooled methanol. The mixture was vortexed and incubated at −20°C for 20 min to facilitate protein precipitation and then centrifuged at 14,000 rpm at 4°C for 15 min. A volume of 400 μL of the supernatant was retrieved and vacuum dried. For MS analysis, reconstitution was performed by adding 100 μL of methanol:water (1:1, v/v), followed by centrifugation at 14,000 rpm at 4°C for 15 min. After completion, the mixture was centrifuged, and the supernatant was collected for analysis. Chromatographic separation was performed using a Thermo Vanquish Flex UHPLC system equipped with a Waters BEH C18 column (2.1 mm × 100 mm). The negative ion mode was used for detection, and quantitative data analysis was carried out with Xcalibur.

#### 2.2.12 Analysis of serum components using UPLC/Q-TOF-MS

A volume of 100 μL of plasma was extracted from the H_AM and control group mice and transferred to 1.5 mL centrifuge tubes. Methanol (300 μL) was added, followed by vortexing for 5 min to precipitate the proteins. The mixture was subsequently centrifuged at 12,000 rpm for 15 min. The supernatant was collected and dried under a nitrogen (N_2_) stream. The residue was dissolved in 300 μL of methanol, vortexed for 2 min, and centrifuged again at 12,000 rpm for 10 min. The supernatant was collected for subsequent analysis. The blood components of the AM extract were also assessed via an UPLC/Q-TOF-MS system. The analytical conditions were identical to those described in section 2.2.2.

#### 2.2.13 Network pharmacological analysis

The active ingredients and corresponding target information of AM were obtained from the Traditional Chinese Medicine System Pharmacology (TCMSP) database (https://tcmspw.com/). The retrieved target data were imported into the UniProt database (https://www.UniProt.org/) for protein ID conversion, enabling the identification of potential gene targets associated with AM. Cytoscape 3.10.0 software was used to construct a network diagram depicting the relationships between the active ingredients of AM and their associated targets. The GeneCards comprehensive database (https://www.genecards.org/) was queried to extract the top 1,500 colitis-related targets. A Venn diagram was generated via Venny 2.1.0 (https://bioinfogp.cnb.csic.es/tools/venny/) to identify overlapping targets between AM targets and colitis-related targets. The intersecting targets were then imported into the STRING database (https://www.stringdb.org/), specifying “*Homo sapien*”’ as the species and applying a minimum confidence score threshold of 0.9. A protein-protein interaction (PPI) network was constructed, and isolated targets were excluded. The resulting TSV file was downloaded for visualization. Cytoscape 3.10.0 was used to create the PPI network diagram and identify the core target proteins. Furthermore, Gene Ontology (GO) functional enrichment and Kyoto Encyclopedia of Genes and Genomes (KEGG) pathway analyses were conducted on the key targets via the Metascape database (https://metascape.org/). The results of the enrichment analysis were visualized in a bubble chart generated through a bioinformatics platform (https://www.bioinformatics.com.cn/).

#### 2.2.14 WB analysis

A 20-fold volume of protein lysis buffer was added to the colon tissue samples (about 20 mg each) for homogenization. The homogenates were incubated at 4°C for 45 min and subsequently centrifuged at 12,000 rpm for 10 min. The supernatant was collected, and protein concentrations were quantified via the bicinchoninic acid (BCA) assay. Proteins were resolved by SDS-PAGE and transferred onto polyvinylidene fluoride (PVDF) membranes. The membranes were blocked for 5 min with a rapid protein-free blocking solution and incubated overnight at 4°C with primary antibodies. After three washes with TBST (10 min each), the membranes were treated with secondary antibodies and incubated for 2 h. Visualization of the protein bands was achieved via enhanced chemiluminescence (ECL) reagents, with *β*-actin serving as the normalization control. The experiments were conducted independently three times, and the data were averaged for subsequent analysis.

#### 2.2.15 Immunofluorescence (IF) analysis

Colon tissues from each group were harvested and embedded in paraffin. After sectioning and antigen retrieval, the sections were blocked with serum to prevent non-specific binding. The primary antibody was applied at the optimal concentration, and the sections were incubated overnight at 4°C. The following day, a fluorescent secondary antibody was applied, and the samples were incubated for 1 h at 37°C in a light-protected, humidified chamber. DAPI was then added to stain the nuclei, which were then incubated for 10 min in the dark. Finally, the sections were mounted with glycerol and immediately examined under a fluorescence microscope.

#### 2.2.16 Statistical analysis

All the data are presented as the means ±standard errors of the means. Group comparisons were performed via one-way analysis of variance (ANOVA). A p value less than 0.05 was regarded as statistically significant, with significance levels denoted as follows: ^*^ *P* < 0.05 and ^**^ *P* < 0.01. The Wekemo bioinformatics cloud (https://www.bioincloud.tech/) was used to perform Spearman correlation analysis via software to obtain correlation heatmaps between microbial communities and their metabolites (SCFAs and BAs).

## 3 Results

### 3.1 Identification of components in AM extract

**FIGURE 1:**
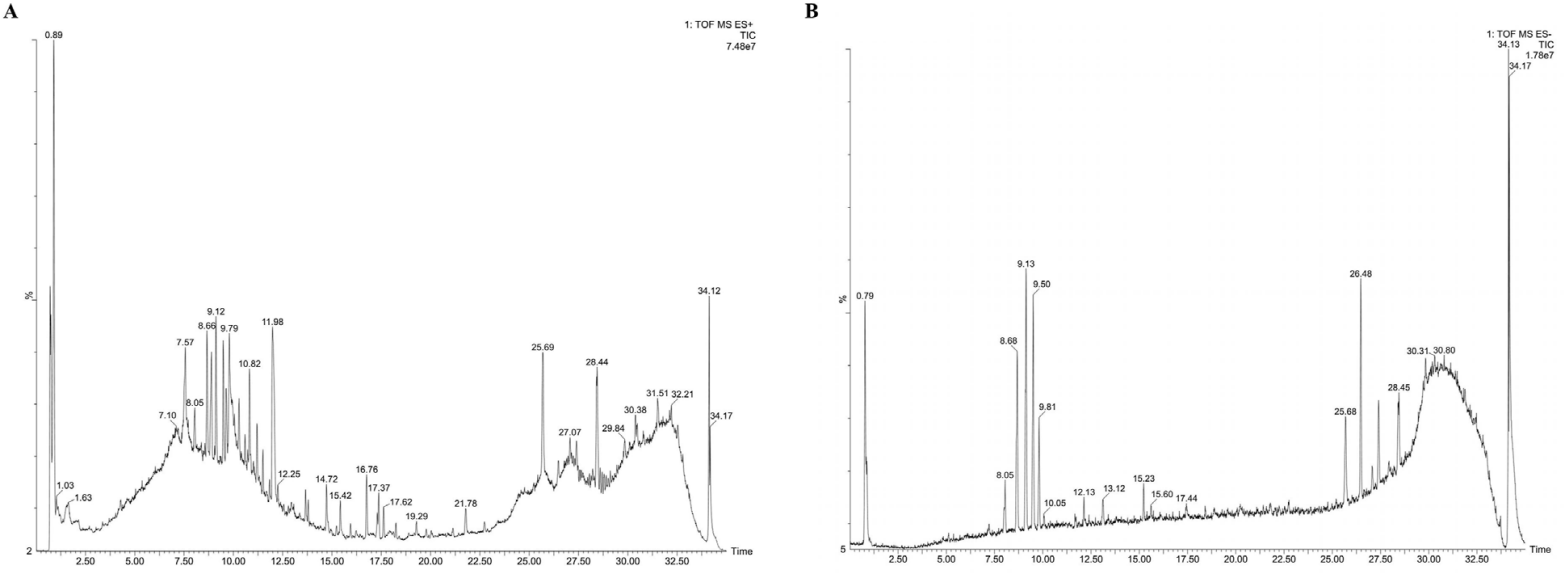
Identification of components in AM extract using UPLC/Q-TOF-MS profiling. **(A)** Positive ion mode; **(B)** Negative ion mode.

High-resolution mass spectrometry data of the AM extract were comprehensively analyzed using UPLC/Q-TOF-MS profiling. Compound identification was performed through database comparison. A total of 45 compounds were identified in the positive ion mode and 40 in the negative ion mode, with characteristic saponins and flavonoids being the major bioactive constituents. The total ion chromatograms for both modes are shown in Figure 1, and detailed identification results are presented in Table 1.

**TABLE 1:** Identification of the chemical constituents of AM extract.

Component name	Identification status	Observed m/z	Observed RT (min)	Response	Adducts
(6aR,11aR)-3-Hydroxy-9,10-dimethoxypterocarpan-3-O-beta-D-glucoside-6″-O-malonate	Identified	549.1607, 549.161	10.76, 11.2	5,759, 185,581	+H, +Na, +K
2-Hydroxy-3-methoxystrychnine	Identified	403.1596, 381.1822	0.9, 0.77	12,252, 6,717	+Na, +H
2′-N-Methyltetrandrinium	Identified	638.338	31.01	14,342	+H
3,9-di-O-Methylnissolin	Identified	337.1079, 315.1256, 337.107, 337.1064, 337.1078, 337.1073, 315.1233	9.95, 18.41, 9.8, 7.93, 9.06, 9.56, 10.68	5,383, 10,972, 7,711, 7,023, 7,823, 5,161, 8,704	+Na, +H
7,2′- Dihydroxy-3′,4′-dimethoxyisoflavan-7-O-β-D- glucoside- 6″-O-malonate	Identified	551.1764	11.5	69,914	+H, +Na, +K
7,2-Dihydroxy-3′,4′-dimethoxyisoflavan	Identified	303.122, 325.1075	10.6, 9.05	49,755, 10,053	+Na, +H
quercetin	Identified	303.2440	21.48	3,785	+H
9,10-Dimethoxypterocarpan-3-O-β-D-glucoside	Identified	463.1603, 465.1754, 487.1611	10.29, 10.6, 9.05	36,331, 13,859, 5,938	+H, +Na, +K
Acetylastragaloside I	Identified	911.502	19.78	9,625	+H, +Na, +K
Adenine	Identified	136.0615	1.12	11,290	+H
Adenine nucleoside	Identified	268.1049, 268.1036	0.89, 1.12	48,734, 21,944	+H
Alexandrin	Identified	615.4049, 599.4237	23.48, 27	8,082, 67,730	+K, +Na
Asernestioside B deglycosylation	Identified	833.4698, 833.4703	16.76, 17.37	18,559, 28,924	+Na
Astragaloside II	Identified	827.4793, 827.4786, 827.4799	14.14, 13.54, 15.43	8,888, 11,363, 22,106	+H, +Na, +K
Astragaloside II deglycosylation	Identified	687.408, 665.4217	16.44, 17.1	9,190, 5,927	+Na, +K, +H
Astragaloside V	Identified	947.5217	11.96	49,349	+H, +Na, +K
Astragaloside VII	Identified	947.5233	13.07	14,809	+H, +Na
AstragalosideIV	Identified	807.4542, 785.4687, 785.4697, 785.4686	14.53, 12.48, 13.8, 13.66	5,198, 27,217, 26,789, 24,844	+H, +Na, +K
Astraisoflavanin	Identified	487.1613, 487.1615	9.79, 7.93	5,574, 11,636	+Na
Astramembranoside A	Identified	815.4791	12.77	11,314	+H
Astramembranoside B	Identified	809.4696, 809.4693, 809.4698	14.73, 15.43, 15.94	32,491, 17,241, 13,123	+Na
beta-Dihydrofucosterol	Identified	453.3449	9.12	12,765	+K
Isorhamnetin	Identified	317.0634	18.90	11,074	+H
Biochanin A	Identified	285.0757, 285.0755, 285.0754, 285.0752	7.55, 8.88, 9.67, 10.73	934,398, 666,150, 234,130, 167,799	+H
Calycosin-7-O-beta-D-glucopyranoside	Identified	447.1293	7.55	1,053,101	+H, +Na, +K
Coumarin	Identified	147.0431	11.5	10,945	+H
Crystal VI	Identified	133.0599	0.79	13,174	+H
Dacarbazine	Identified	205.0822	0.9	14,241	+Na
Foliosidine	Identified	308.1497	0.9	10,028	+H
Formononetin 7-O-Glucoside	Identified	431.134	9.63	564,083	+H, +Na, +K
Glycogen	Identified	705.1852	0.76	16,248	+K, +Na
Hederagenin	Identified	473.3621, 473.3626, 473.3627, 473.3624, 473.3618	23.8, 26.73, 28.76, 23.67, 25.43	16,096, 17,249, 23,982, 25,965, 10,525	+H
Isoastragaloside I	Identified	869.4914, 907.4483, 869.4911, 869.4924, 869.491,	16.76, 6.83, 15.25, 18.24, 17.37, 15.84	148,572, 8,230, 11,567, 13,398, 106,315	+H, +Na, +K
Isoastragaloside II	Identified	827.4805, 827.4801	14.72, 15.94	64,377, 11,728	+H, +Na, +K
Jaranol	Identified	315.0864, 315.0862, 315.0862	8.96, 7.75, 18.06	7,850, 12,432, 33,687	+H
L-Arginine	Identified	175.1179, 175.1176, 175.1181	1.26, 0.92, 0.74	5,340, 18,321, 254,460	+H, +Na, +K
L-Canavanine	Identified	177.0972, 177.0966, 177.0985	0.9, 1.17, 4.98	27,661, 9,157, 8,176	+H
Lup-20(29)-en-3-one	Identified	447.3553	7.55	39,935	+Na
Methylnissolin	Identified	301.1071, 301.1066, 301.106, 301.1067	12.26, 11.2, 10.76, 10.29	113,825, 265,581, 11,144, 433,952	+H
Proline	Identified	116.07	0.81	45,270	+H
Methylnissolin	Identified	301.1071	12.26	113,825	+H
Rhamnocitrin	Identified	301.0702, 301.0706, 301.0707, 301.0681, 301.0699	8.76, 7.62, 9.96, 11.47, 6.64	41,967, 12,111, 39,023, 8,647, 26,260	+H
Rhamnocitrin 3-O-glucoside	Identified	463.1238	8.76	45,576	+H
Soyasaponin I	Identified	943.5278	14.76	148,133	+H, +Na, +K
Terfenadine	Identified	472.3183	9.5	10,839	+H
(6aR,11aR)-3-Hydroxy-9,10-dimethoxypterocarpan-3-O-beta-D-glucoside-6″-O-malonate	Identified	593.1534	9.13	17,935	+HCOO
(6aR,11aR)-3-Hydroxy-9,10-dimethoxypterocarpan-3-O-beta-D-glucuronide	Identified	475.1262	9.64	677,184	−H
(S)-Coriolic acid	Identified	295.2279	20.25	20,847	−H
(Z,Z,Z)-9,12,15-Octadecatrienoic acid	Identified	277.2183	24.6	27,711	−H
20(R)-21,24-Cyclo-3beta,25-dihydroxyldammar-23(24)-en-21-one	Identified	455.3535, 455.3542	30.13, 30.49	23,885, 84,984	−H
7,2′- Dihydroxy-3′,4′-dimethoxyisoflavan-7-O-β-D- glucoside- 6″-O-malonate	Identified	549.1634, 549.1633, 549.1642, 549.1639, 549.1635, 549.1639, 301.1089	11.51, 11.71, 11.35, 11.2, 12.27, 12.48, 10.61	13,005, 14,309, 22,412, 61,365, 209,226, 10,614, 150,314	−H
7,2′-Dihydroxy-3′,4′-dimethoxyisoflavone	Identified	313.0727	8.97	15,375	−H
9,10-Dimethoxy-pterocarpan-3-O-β-D-glucopyranoside	Identified	507.1517, 509.168, 463.1624	10.3, 10.5, 10.61	770,597, 26,572, 381,784	+HCOO, −H
Acetylastragaloside	Identified	955.4926, 909.4855, 955.4933, 955.4941	19.79, 17.63, 14.64, 16.32	214,784, 22,771, 51,627, 26,153	+HCOO, −H
Alexandrin	Identified	621.4382	30.61	521,227	+HCOO, −H
Astragaloside II deglycosylation	Identified	709.418	16.45	15,831	+HCOO
Astragaloside III	Identified	829.4602, 829.461, 829.462, 829.4617	13.68, 13.81, 12.48, 13.34	1,044,483, 572,879, 63,402, 19,640	+HCOO, −H
Astragaloside IV deglycosylation	Identified	667.407	14.82	67,444	+HCOO
Astragaloside V	Identified	991.5157, 991.5155, 991.5135	13.08, 11.67, 12.77	215,504, 12,221, 11,527	+HCOO, −H
Astragaloside VI	Identified	991.5155	11.96	137,961	+HCOO, −H
Astramembranoside A	Identified	859.4725, 859.4703, 859.4729	12.33, 12.78, 12.47	13,708, 15,079, 19,647	+HCOO, −H
Astramembranoside B	Identified	831.4755, 831.4769, 831.4764	12.8, 12.39, 12.68	65,042, 44,027, 45,836	+HCOO, −H
Astrasieversianin XV	Identified	945.5085	11.94	15,492	+HCOO
Biochanin A	Identified	283.0623, 283.0621, 283.0621, 283.0623, 283.062, 283.0623	12.26, 9.68, 8.71, 10.74, 8.56, 8.89	34,035, 95,658, 120,937, 129,346, 183,824, 1,399,880	−H
Calycosin 7-O-glucoside	Identified	491.1207	7.57	1,166,148	+HCOO, −H
Crystal VI	Identified	131.0464	0.76	40,248	−H
D-(+)-Sorbose	Identified	179.0566	0.81	43,271	−H
D-Glucuronic Acid	Identified	193.0357	0.79	11,418	−H
D-Mannuronic acid	Identified	195.0516	0.78	60,847	−H
Docosanoic acid	Identified	339.3274	33.62	16,061	−H
Glycogen	Identified	711.2158	9.17	49,003	+HCOO, −H
Hesperetic acid	Identified	193.051, 239.0571, 193.0511, 193.0509	6.65, 5.41, 7.24, 6.55	14,637, 166,906, 37,245, 10,222	−H, +HCOO
Isoastragaloside I	Identified	913.4805, 913.4822, 913.4833, 913.4829	16.77, 17.38, 18.25, 15.26	2,020,583, 1,113,463, 332,808, 32,706	+HCOO, −H
Isoastragaloside II	Identified	871.4727, 871.4716, 871.4714, 871.4718, 871.4712, 871.4718, 871.4713	15.95, 15.44, 14.73, 13.55, 14.39, 14.15, 13.46	285,148, 610,359, 1,253,934, 41,404, 31,069, 15,046, 21,913	+HCOO, −H
Isomucronulatol-7,2′-di-O-glucoside	Identified	625.2147, 671.2193	9.49, 8.53	10,443, 30,247	+HCOO, −H
L-Arginine	Identified	173.1048	0.75	11,679	−H
Methylnissolin	Identified	299.0929, 299.0939, 299.0942	10.3, 11.36, 11.21	33,835, 23,298, 579,506	−H
Rhamnocitrin	Identified	299.0572, 299.0572, 299.0568	9.97, 8.77, 9.77	146,515, 43,805, 12,637	−H
Rhamnocitrin 3-O-glucoside	Identified	507.1162, 461.1106, 507.1162	8.77, 6.65, 8.77	49,252, 29,170, 49,252	+HCOO, −H
Soyasaponin I	Identified	987.5195	14.77	276,690	+HCOO, −H
Soyasaponin II	Identified	911.5021	14.74	39,363	−H, +HCOO
Soyasaponin II	Identified	957.5083	11.76	14,512	+HCOO
Syringaresinol	Identified	417.1545, 417.1548	8.56, 9.19	12,266, 11,877	−H, +HCOO
3,9-di-O-Methylnissolin	Identified	313.1093, 313.1098, 313.1085, 313.1088	10.17, 8.86, 9.45, 9.9	1,544, 759, 4,915, 6,263	−H
Hederagenin	Identified	471.3484, 471.3476, 471.3483	20.47, 27.7, 28.77	415, 1,666, 2,289	−H
Isorhamnetin	Identified	315.0506, 315.049, 315.0504, 315.0487	9.18, 18.25, 18.91, 7.91	1,501, 336, 455, 785	−H

### 3.2 HPLC analysis

**FIGURE 2:**
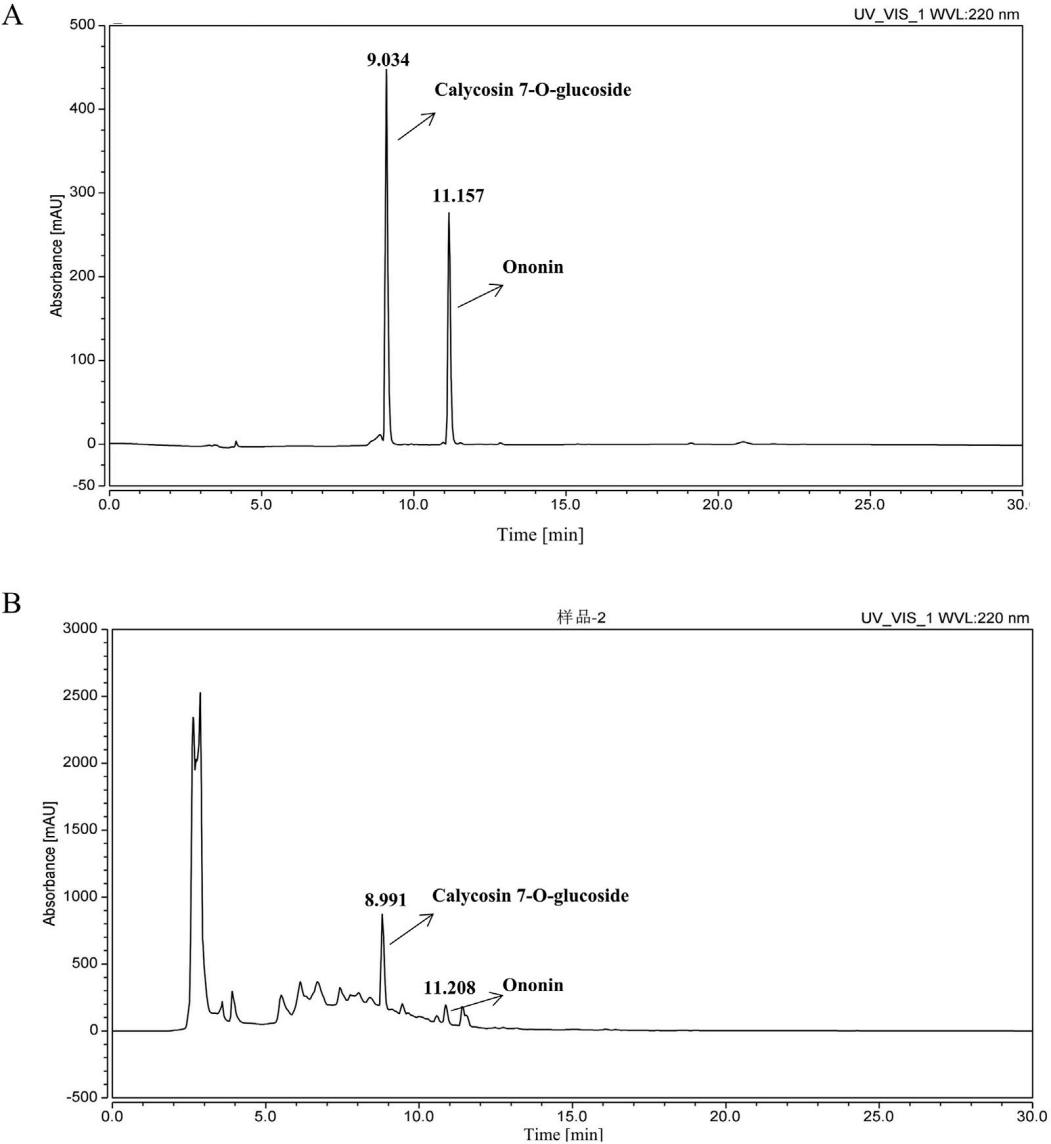
HPLC chromatogram. **(A)** mixed reference; **(B)** AM extract sample.

Figure 2 presents the HPLC chromatogram. Prior to administering the AM extract to UC mice, the principal constituents were quantitatively analyzed using the HPLC method. Based on retention time comparisons with individual standards, calycosin-7-O-glucoside and ononin were identified in ascending order of peak appearance. The concentrations of these compounds were determined to be 0.68 mg/g and 0.1245 mg/g.

### 3.3 AM extract alleviated colitis symptoms in UC mice

**FIGURE 3:**
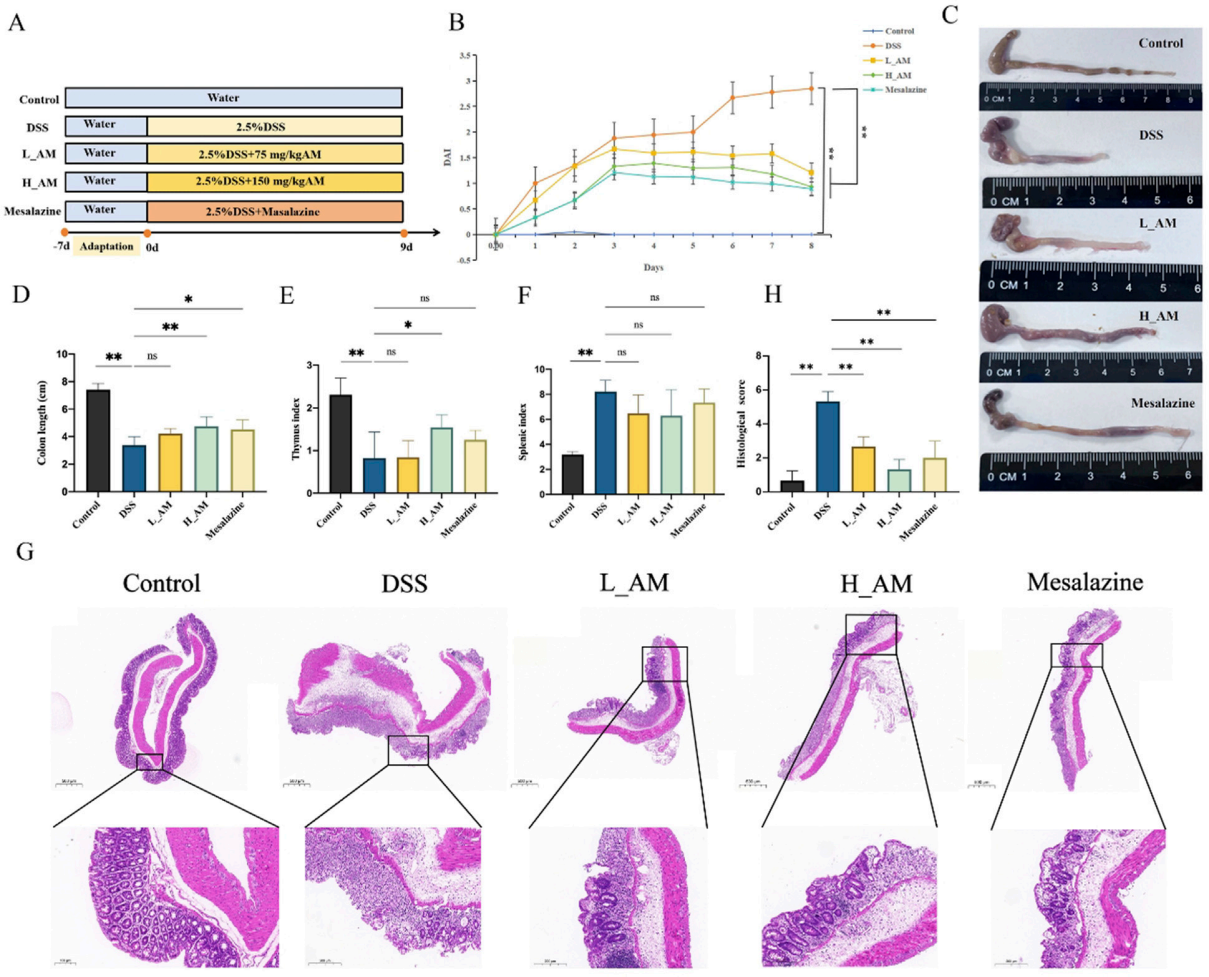
AM extract alleviates colitis symptoms in UC mice. **(A)** experimental workflow diagram. **(B)** DAI scores. **(C)** Representative images of the colon. **(D)** Colon length. **(E,F)** Thymus and Spleen indexe. **(G,H)** Representative images of H&E stainings. **(H)** HE staining score. The data are expressed as mean ± SEM. (n = 7 in **(B,D–F)** n = 3 in **(G,H)**. *P < 0.05, **P < 0.01.

Figure 3A illustrates the workflow of the experimental procedure. As depicted in Figure 3B, the DAI score of the DSS-treated group was significantly greater than that of the control group (*P* < 0.01). Treatment with AM extract markedly reduced the DAI score (*P* < 0.01), indicating effective alleviation of symptoms such as weight loss, abnormal stool consistency, and hematochezia. Furthermore, AM extract improved DSS-induced colon shortening in a dose-dependent manner (*P* < 0.01) (Figures 3C,D). UC mice presented a reduced thymus index and increased spleen index, whereas the AM extract significantly restored the thymus index (*P* < 0.05) (Figures 3E,F). Additionally, H&E staining revealed intact colon mucosa in the control group, with well-preserved crypt structures and an abundance of goblet cells. In contrast, the DSS group exhibited significant inflammatory cell infiltration, ulcer formation, and severe damage to crypt structures, goblet cells, and epithelial tissue. Treatment with AM extract in both the L_AM and H_AM groups ameliorated these pathological changes by reducing mucosal ulcers and inflammatory cell infiltration (Figures 3G,H). These findings suggest the potential of AM extract to ameliorate UC-related damage, with the H_AM group showing an anti-inflammatory effect comparable to that of mesalazine.

### 3.4 AM extract regulated intestinal microbiota diversity in UC mice

**FIGURE 4:**
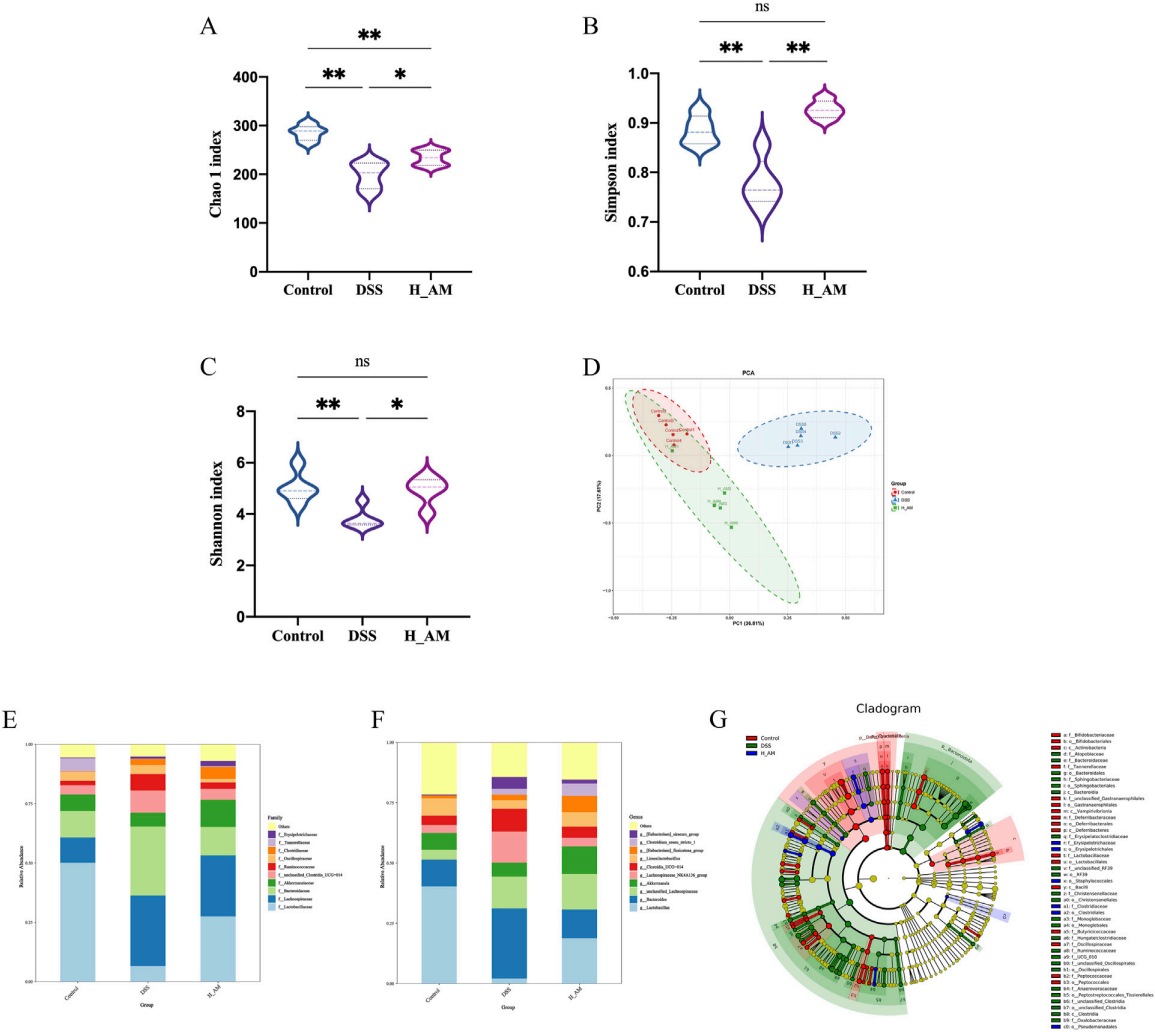
AM extract improves intestinal barrier permeability. **(A–C)** The ɑ diversity indexes of Chao1, Simpson, and Shannon. **(D)** PCA score plot. **(E,F)** Bar plot of community composition at the family level and genus level. The data are expressed as mean ± SEM. **(G)** Differences in dominant microorganisms among three groups on the basis of a cladogram. (n = 5 in **(A–G)**. *P < 0.05, **P < 0.01.

The gut microbiota is essential for the onset of UC. In UC patients, dysbiosis of the gut microbiota is observed, characterized by decreased microbial diversity and an imbalance between commensal and harmful bacteria. This disturbance compromises the intestinal barrier, resulting in greater gut permeability and a worsening of inflammation (Ren et al., 2025). As shown in Figures 4A–C, the DSS group presented notable reductions in *α* diversity indices, including the Chao1, Simpson, and Shannon indices, compared with those of the control group (*P* < 0.01). Conversely, the H_AM group presented a significant improvement in these diversity indices relative to those of the DSS group (*P* < 0.05 or *P* < 0.01), indicating that the AM extract promoted the restoration of gut microbiota diversity. The principal component analysis (PCA) results (Figure 4D) clearly revealed a separation between the control and DSS groups, reflecting significant changes in microbial profiles due to DSS-induced dysbiosis. The H_AM group, while distinct from both the control and DSS groups, was closer to the control group, suggesting that the AM extract promoted a microbiota profile similar to that of the healthy state.

Alterations in gut microbiota composition—particularly reductions in the abundance of specific beneficial genera—are recognized as key contributors to the pathogenesis of IBD (Haneishi et al., 2023). Analysis of microbial relative abundance at the family level (Figure 4E) revealed that the Control group was primarily composed of *Lactobacillus*, Lachnospiraceae, and Bacteroidaceae. In contrast, the DSS group exhibited a marked reduction in *Lactobacillus* and Akkermansiaceae, accompanied by an increase in Lachnospiraceae. Treatment with AM extract reversed these dysbiotic changes, as evidenced by the H_AM group, which displayed elevated relative abundance of *Lactobacillus* and Akkermansiaceae, along with reduced levels of Lachnospiraceae. At the genus level (Figure 4F), AM extract significantly enhanced the relative abundance of *Lactobacillus* and *Akkermansia*, both of which were decreased in the DSS group. LEfSe analysis (Figure 4G) further demonstrated that taxa belonging to the family Lactobacillaceae were significantly enriched in the Control group. In contrast, taxa such as family Bacteroidaceae, family Deferribacteraceae and family Tannerellaceae were notably more abundant in the DSS group, suggesting a potential association with gut inflammation or microbial dysbiosis. Additionally, the H_AM group was distinguished by enrichment of Clostridiaceae and Erysipelotrichaceae, suggesting their potential role in promoting gut health or contributing to the re-establishment of microbial balance. These findings underscore the modulatory effect of AM extract on gut microbiota, highlighting its capacity to increase beneficial bacteria while suppressing potentially pathogenic taxa, thereby exerting a protective effect against UC.

### 3.5 AM extract corrected SCFAs metabolism in UC mice

**FIGURE 5:**
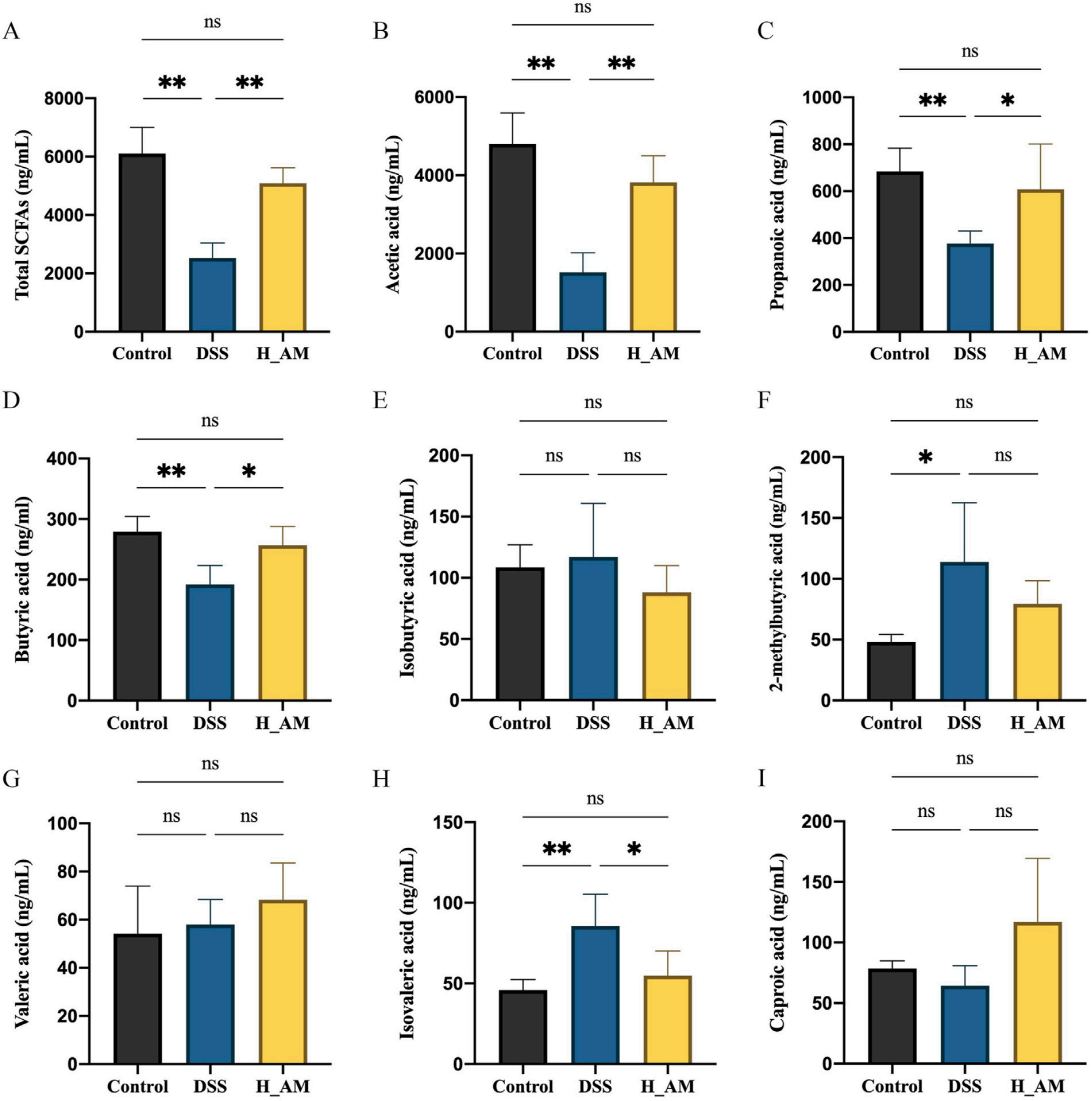
AM extract regulates SCFAs metabolism in plasm of UC mice. **(A–I)** The contents of Total SCFAs, acetic acid, Propionic acid content, Butyric acid content, Isobutyric acid content, 2-methybutyric acid content, Valeric acid content, Isovaleric acid content, Caproic acid content. The data are expressed as mean ± SEM, (n = 5 in **(A–I)**. *P < 0.05, **P < 0.01.

SCFAs, including acetate, propionate, and butyrate, are critical metabolites for maintaining gut homeostasis (Du et al., 2024). IBD patients often exhibit reduced levels of SCFAs due to a decrease in SCFAs-producing bacteria in mucosal tissues and feces (Parada Venegas et al., 2019). As shown in Figure 5, the DSS group presented significantly lower serum levels of acetic acid, propionic acid, and butyric acid than the control group (*P* < 0.01), along with a marked increase in isovaleric acid levels (*P* < 0.01). Treatment with AM extract significantly restored the levels of acetic acid, propionic acid, and butyric acid while reducing the levels of isovaleric acid (*P* < 0.01 or *P* < 0.05). These results suggest that the AM extract effectively modulates SCFA metabolism, which likely contributes to the protection of the mucosal barrier in UC mice.

### 3.6 AM extract corrected BAs metabolism in UC mice

**FIGURE 6:**
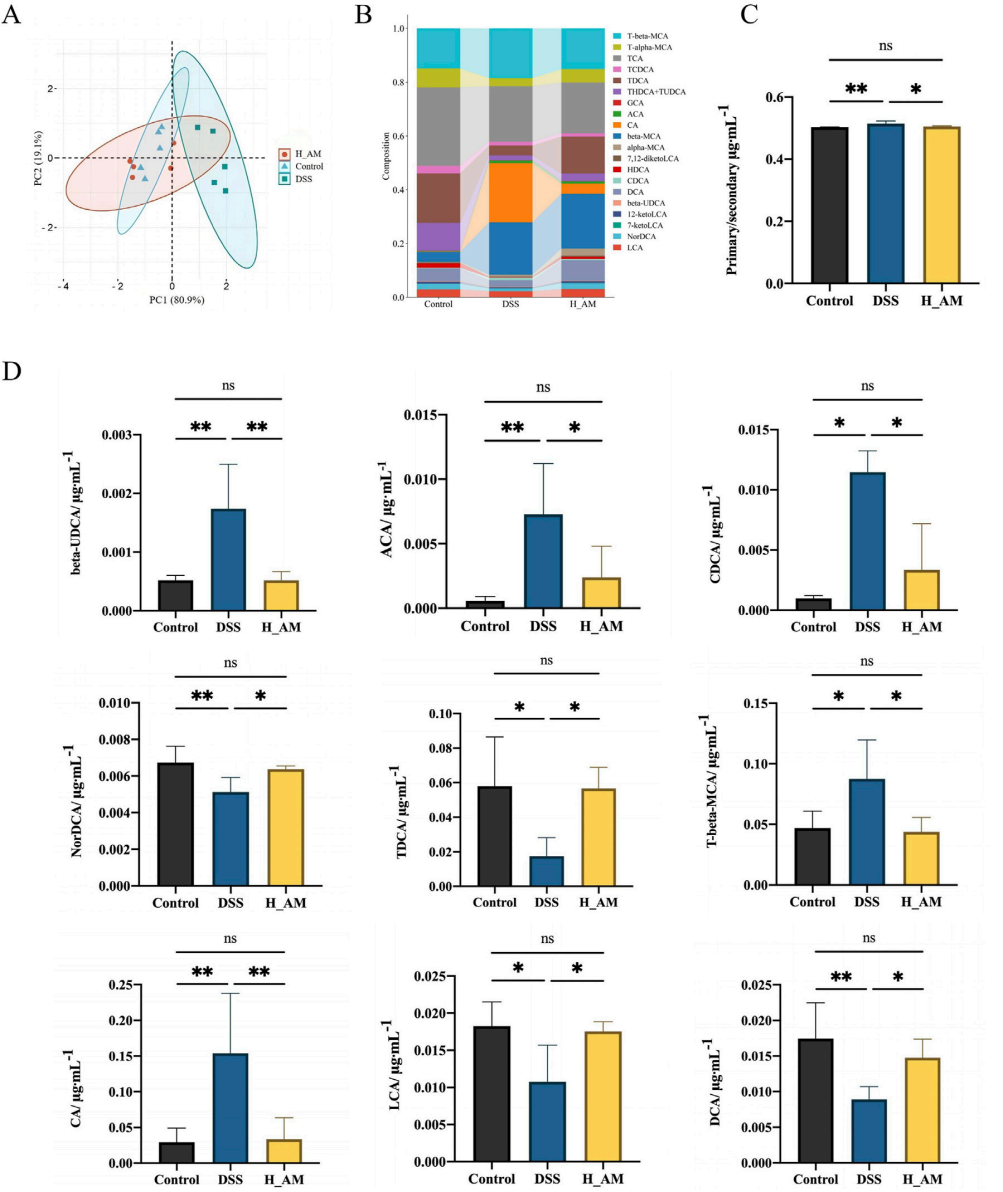
AM extract regulates BAs metabolism in plasm of UC mice. **(A)** PCA analysis. **(B)** BAs composition. **(C)** Ratio of primary BAs to secondary Bas. **(D)** The contents of beta-UDCAs, ACA, CDCA, NorDCAs, TDCAs, T-beta-MCA, CA, LCA, DCA. The data are expressed as mean ± SEM. (n = 5 in **(A–D)**. *P < 0.05, **P < 0.01.

BAs, key metabolites of the gut microbiota, are intricately linked to intestinal inflammation. In this study, absolute quantification via an LC-MS platform identified 20 BAs from a pool of 39 detected metabolites. The PCA plot (Figure 6A) revealed that the serum BAs metabolic profiles of the control and H_AM groups clustered closely, whereas those of the DSS group were distinctly separated into different quadrants. These findings suggested that AM extract significantly altered serum BAs metabolism in UC mice, which aligns the metabolic profile of the H_AM group more closely with that of the control group and thereby restores a healthy BAs metabolic phenotype.

The BAs composition as shown in Figure 6B. The ratio of primary BAs to secondary BAs, a marker of metabolic dysregulation, was elevated in the DSS group compared with the control group (*P* < 0.01) (Figure 6C). AM extract treatment significantly reduced this ratio, which approached the levels observed in the control group (*P* < 0.05). These findings indicate that the AM extract promotes the conversion of primary BAs to secondary BAs, thereby mitigating metabolic disturbances. Quantitative analysis (Figure 6D) further revealed elevated levels of *β*-UDCA, ACA, CDCA, T-*β*-MCA, and CA in the DSS group, with reduced levels of norDCA, TDCA, LCA, and DCA (*P* < 0.05 or *P* < 0.01). Treatment with AM extract effectively reversed these changes *(P* < 0.05 or *P* < 0.01), notably increasing the levels of DCA and LCA, which act as agonists of Takeda G protein-coupled receptor 5 (TGR5) and farnesoid X receptor (FXR). TGR5 and FXR are pivotal in maintaining intestinal barrier integrity and exerting anti-inflammatory effects (Nie et al., 2024; Sorrentino et al., 2020; Vavassori et al., 2009).

### 3.7 Corrections analysis

**FIGURE 7:**
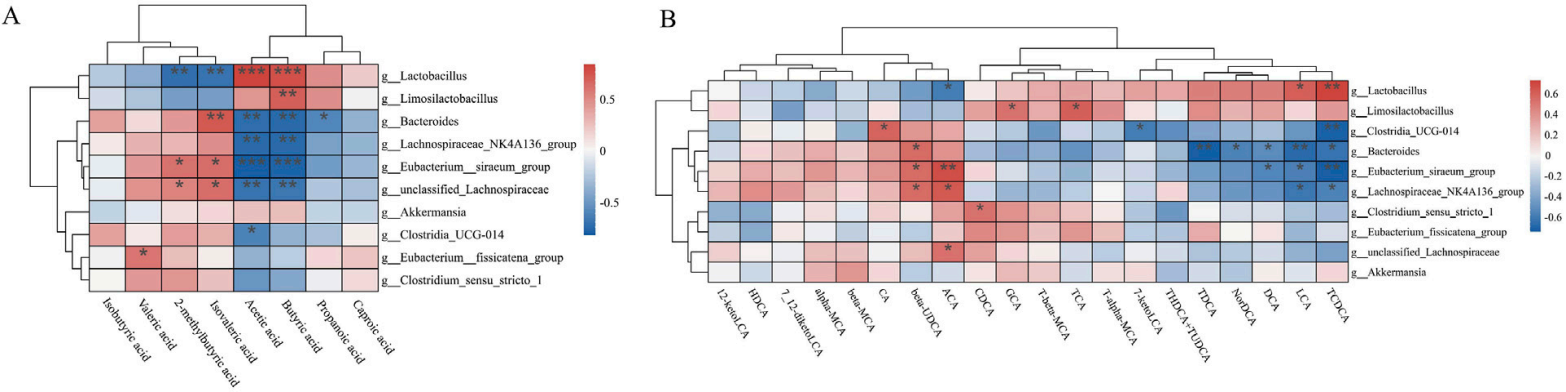
Spearman correlation analysis of bacterial genera and SCFAs **(A)** and BAs **(B)**.

Given the well-established interdependence between microbial communities and metabolites, we performed Spearman correlation analysis to investigate the associations between differential gut microbiota and metabolites. As illustrated in Figure 7A, significant positive correlations were observed between the genus *Lactobacillus* and several SCFAs, including acetic acid and butyric acid (*P* < 0.001), suggesting that *Lactobacillus* may promote the production or accumulation of these specific SCFAs. This observation aligns with the earlier LEfSe results. In contrast, the Eubacterium_siraeum_group exhibited significant negative correlations with both acetic acid and butyric acid (*P* < 0.001). Furthermore, Spearman correlation analysis was also conducted between bacterial genera and BAs (Figure 7B). Both *Lactobacillus* and the Eubacterium_siraeum_group demonstrated positive correlations with TCDCA, ACA, LCA, and β-UDCA levels (*P* < 0.001 or *P* < 0.05), while also showing negative correlations with ACA, DCA, LCA, and TCDCA levels (*P* < 0.001 or *P* < 0.05). Notably, *Bacteroides* exhibited significant negative correlations with TCDCA, LCA, DCA, TDCA, and NorDCA (*P* < 0.001 or *P* < 0.05), suggesting that these bacterial taxa may exert inhibitory or regulatory effects on the metabolism or synthesis of these BAs. Collectively, these findings suggest that the protective effect of AM extract against UC may be closely linked to its modulatory influence on gut microbiota composition and associated metabolites, particularly SCFAs and BAs.

### 3.8 AM extract repaired intestinal barrier injury in UC model mice

**FIGURE 8:**
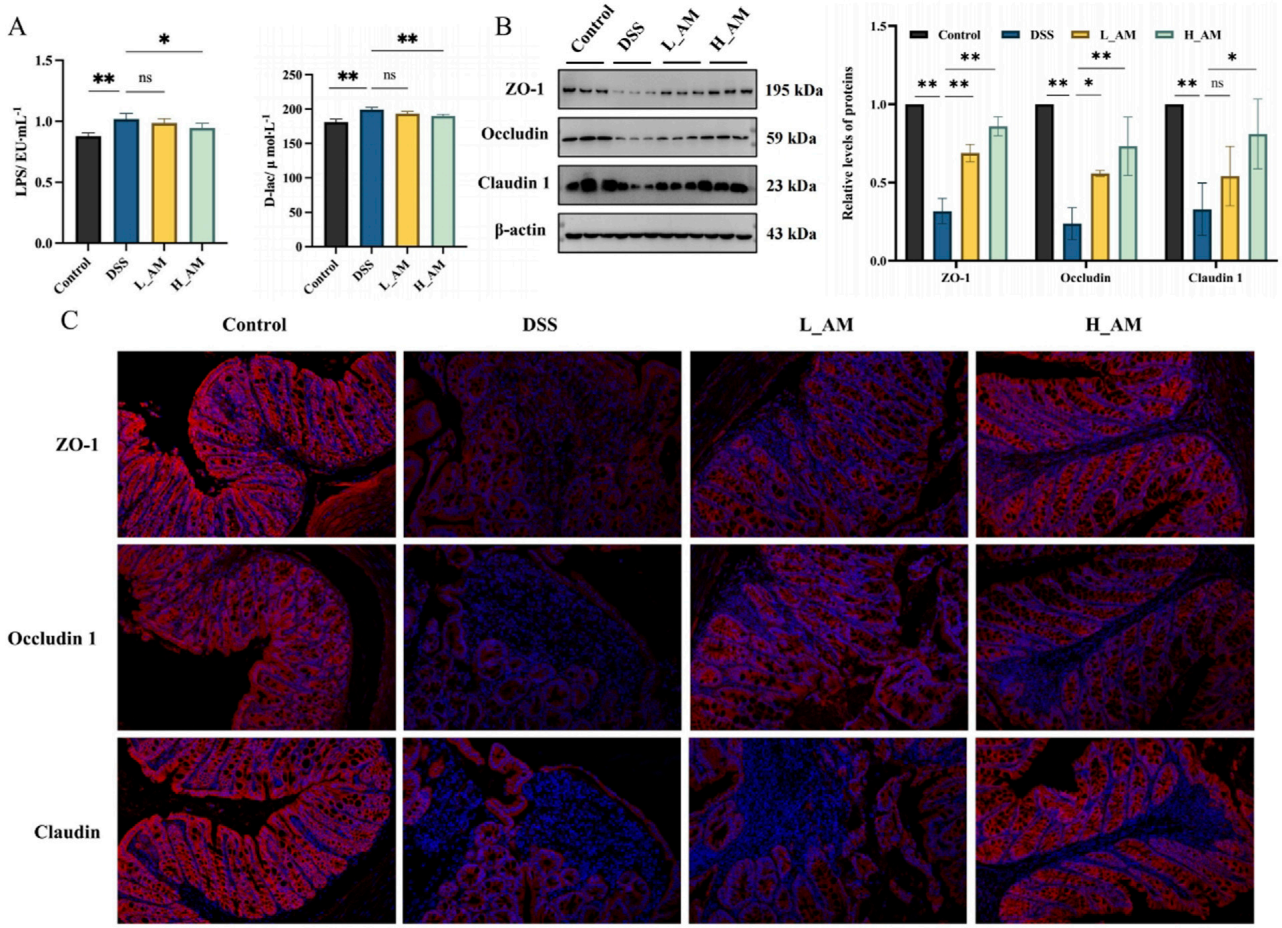
Effect of AM on the intestinal barrier damage. **(A)** LPS and D-lac levels in the serum. **(B)** The protein expressions of ZO-1, Occludin, and Claudin 1 in colon tissues. **(C)** The fluorescence intensity of ZO-1, Occludin, and Claudin 1 in colon tissues. The data are expressed as mean ± SEM. (n = 5 in A, B; n = 3 in **(C)**. *P < 0.05, **P < 0.01.

The compromise of the intestinal barrier can lead to adverse effects such as immune dysfunction and inflammation. Blood levels of LPS and D-lac serve as indicators of changes in intestinal mucosal permeability and the extent of barrier damage (Schoultz and Keita, 2020; Wang et al., 2023). As shown in Figure 8A, the DSS group presented significantly greater LPS and D-lac levels than the Control group (*P* < 0.01), indicating impaired barrier function. Treatment with AM extract, particularly in the H_AM group, resulted in a notable reduction in these levels (*P* < 0.05 or *P* < 0.01), suggesting that AM extract improves intestinal mucosal permeability and reduces barrier damage. Additional Western blotting and immunofluorescence analyses revealed that AM extract notably upregulated the protein expression of ZO-1, Occludin, and Claudin 1 (*P* < 0.05 or *P* < 0.01) (Figures 8B,C). These findings collectively suggest that AM extract plays a significant role in repairing DSS-induced intestinal barrier damage.

### 3.9 AM extract reduced inflammation in UC mice

**FIGURE 9:**
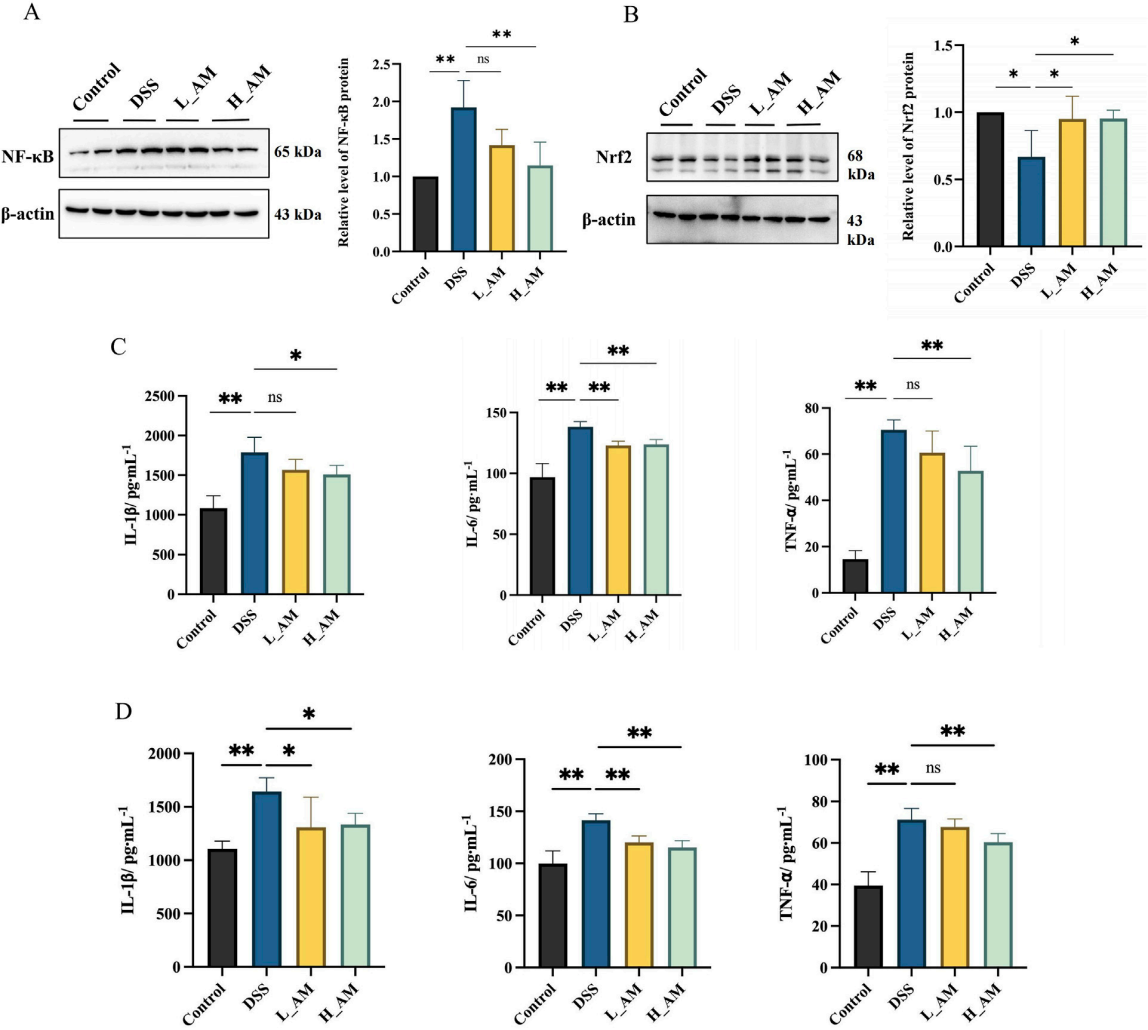
AM relieves inflammatory responses. **(A)** The protein expressions of NF-κB. **(B)** The protein expressions of Nrf2. **(C)** Levels of IL-1*β*, IL-6 and TNF-*α* in colon tissues. **(D)** Levels of IL-1*β*, IL-6 and TNF-*α* in serum. The data are expressed as mean ± SEM. (n = 3 in A, B; n = 6 in **(C)**. *P < 0.05, **P < 0.01.

The gut microbiota is a highly complex microbial ecosystem, and its disruption can significantly impair the intestinal mucosal barrier, often leading to inflammation. These findings indicate that an imbalance in the gut microbiota is a major factor contributing to UC pathogenesis (Quaglio et al., 2022). Therefore, further clarification of the anti-inflammatory effects of AM extract is necessary. First, we observed a significant increase in the relative expression of NF-κB and a significant decrease in the relative expression of Nrf2 in the DSS group compared with those in the control group (*P* < 0.05 or *P* < 0.01) (Figures 9A,B). Similarly, Figure 9C,D clearly show that the levels of IL-1*β*, IL-6, and TNF-*α* were significantly elevated in the colon and serum of DSS-induced colitis mice compared with those in the control group (*P* < 0.01). However, intervention with AM extract effectively attenuated the increase in these inflammatory markers (*P* < 0.05 or *P <* 0.01), suggesting that AM extract may have exerted a significant anti-inflammatory effect on the mice with colitis.

### 3.10 Identification of blood components

**FIGURE 10:**
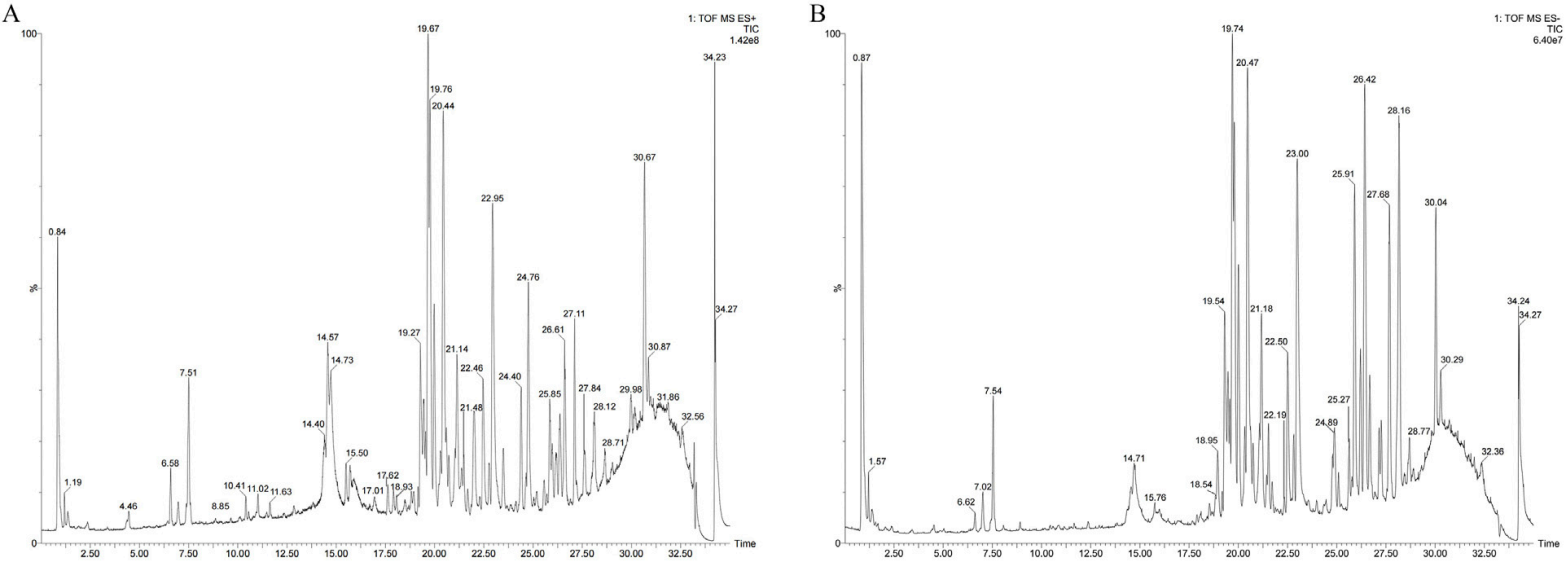
Identification of blood components using UPLC/Q-TOF-MS profiling. **(A)** Positive ion mode; **(B)** Negative ion mode.

Concurrently, a search of the TCMSP database identified 90 compounds associated with AM. Using the criteria of OB ≥ 30% and DL ≥ 0.18, 21 active ingredients were determined to meet the threshold (Wang et al., 2022). UPLC/Q-TOF-MS analysis of plasm in both positive and negative ion modes (Figures 10A,B) identified 113 chemical components on the basis of quasimolecular ion data and secondary fragments. On this basis, we identified five chemical components in the blood that originated from AM through analysis and comparison (Table 2).

**TABLE 2:** Identification of the chemical constituents of the blood components.

Component name	Observed RT (min)	Identification status	Observed m/z	Response	Adducts	Total fragments found
Jaranol	8.85, 18.83, 25.86	Identified	315.0860, 337.0712, 315.0888	860, 1,323, 3,005	+H, +Na	16, 5, 37
Hederagenin	28.31, 27.44, 27.84, 28.71	Identified	473.3607, 473.3598, 473.3626, 473.3625	1,245, 1,304, 3,249, 4,232	+H, +Na	1, 10, 26
3,9-di-O-Methylnissolin	21.28, 18.25, 17.01, 18.36	Identified	353.0819, 314.1154	729, 1,101, 1,593, 45,603	+K, +H, +Na	3, 1, 4
Isorhamnetin	18.93	Identified	317.0634	11,074	+H	9
quercetin	21.48	Identified	303.2440	3,785	+H	2
Isorhamnetin	18.54, 18.95, 18.79, 22.19, 19.74, 18.34, 19.54, 20.30	Identified	315.0495, 315.0492, 315.0496, 315.0484, 315.049, 315.0497, 315.049	8,736, 33,065, 4,342, 2,708, 1,374, 1,239, 1,061, 1,046	−H	4, 3, 2, 5
Hederagenin	27.92, 28.77, 25.27, 22.48, 28.95, 28.36	Identified	471.3467, 471.3474, 471.3482, 471.349, 471.3445, 471.34722	5,598, 4,756, 4,206, 1862, 1,276, 2,471	−H	3, 1, 14, 9
3,9-di-O-Methylnissolin	1.57	Identified	359.1102	356	+HCOO	1

### 3.11 Network pharmacology analysis

#### 3.11.1 Target acquisition of AM and enteritis

**FIGURE 11:**
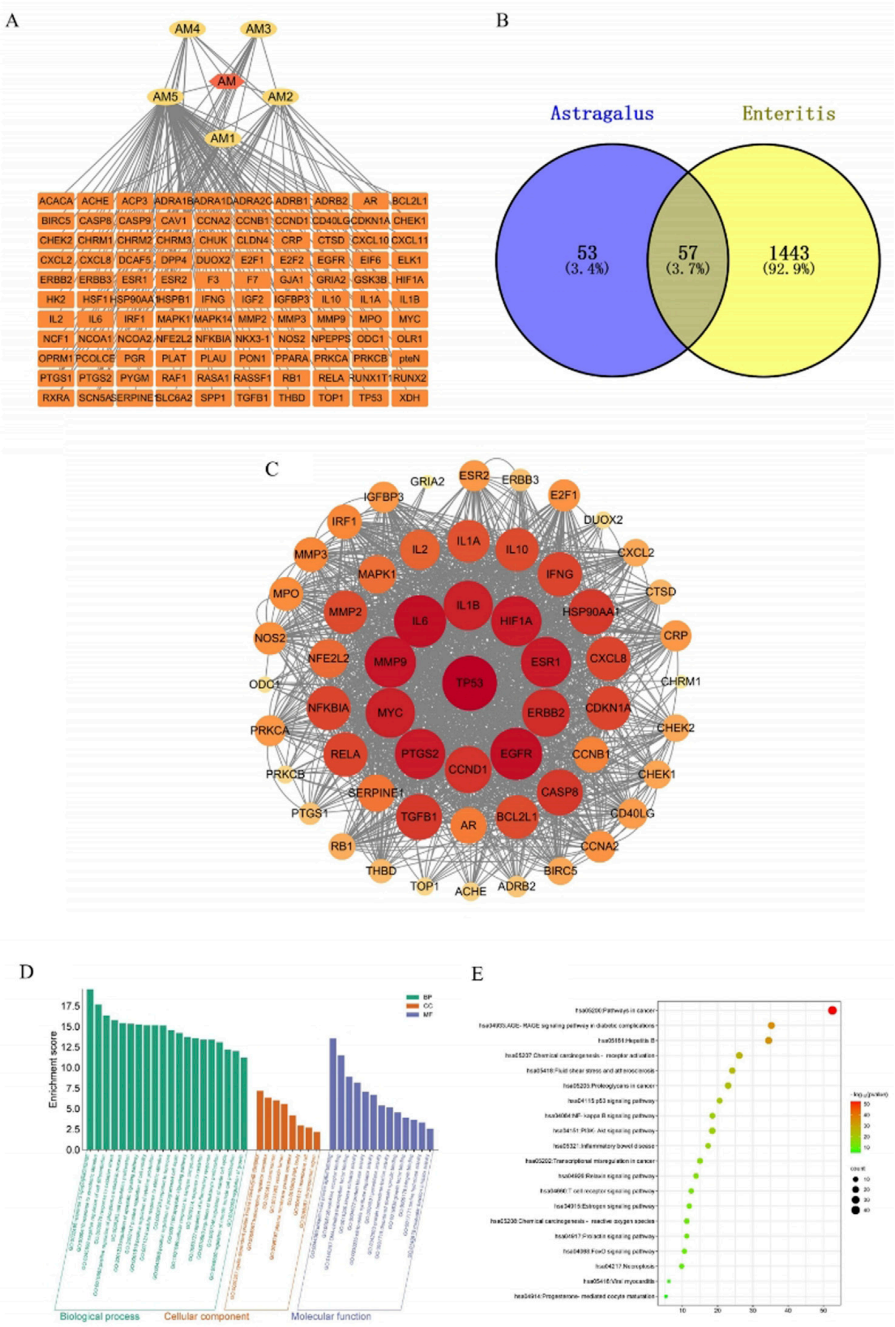
Network pharmacology analysis. **(A)** AM-Potential Pharmacodynamic targets networks. (Yellow represents the active ingredient of AM, while the orange represents potential pharmacodynamic targets) **(B)** Intersection-targets of AM-related targets and Enteritis-related targets. **(C)** PPI networks. ((From red to yellow (or from large to small) indicates that the degree of correlation decreases gradually). **(D)** GO function enrichment analysis. **(E)** KEGG pathway enrichment analysis. (The bubble size indicates the number of genes enriched in each pathway. From green to red indicates that the degree of correlation increases gradually).

Target protein information for these active ingredients was retrieved from the TCMSP database and converted into protein IDs using the UniProt database, resulting in the identification of 110 gene targets associated with AM. The targets associated with the AM extract were visualized via Cytoscape 3.10.0 software to construct a network diagram (Figure 11A), where yellow nodes represent active ingredients and orange nodes denote their respective targets. A total of 9,725 inflammatory disease-related targets were obtained from the GeneCards database, and the top 1,500 acute enteritis targets were selected. Mapping of the 133 AM-associated gene targets to these 1,500 enteritis-related targets revealed 57 overlapping targets potentially relevant to DSS-induced enteritis treatment with AM. These overlapping targets are illustrated in a Venn diagram (Figure 11B).

#### 3.11.2 PPI network analysis

A total of 53 potential targets were input into STRING for the analysis of protein interactions, resulting in the construction of a protein interaction network. This network consisted of 57 nodes and 777 edges, with an average node degree of 27.3 and an average local clustering coefficient of 0.777. The PPI networks were screened using Cytoscape 3.10.0 (Figure 11C). In this network, darker colors represent higher target degree values. The core target proteins identified for the treatment of enteritis with AM include TP53, IL6, and IL1B, among others.

#### 3.11.3 Signaling pathway and functional enrichment analysis

GO enrichment analysis via the Metascape database revealed 20 pathways related to biological processes (BP), 8 pathways associated with cellular components (CC), and 13 pathways associated with molecular functions (MF) (Figure 11D). Additionally, KEGG pathway enrichment analysis (Figure 11E) revealed 20 signaling pathways, notably the PI3K/AKT signaling pathway, which may play crucial roles in the therapeutic effects of AM extract.

### 3.12 AM extract upregulated the PI3K/AKT signaling pathway in UC mice

**FIGURE 12:**
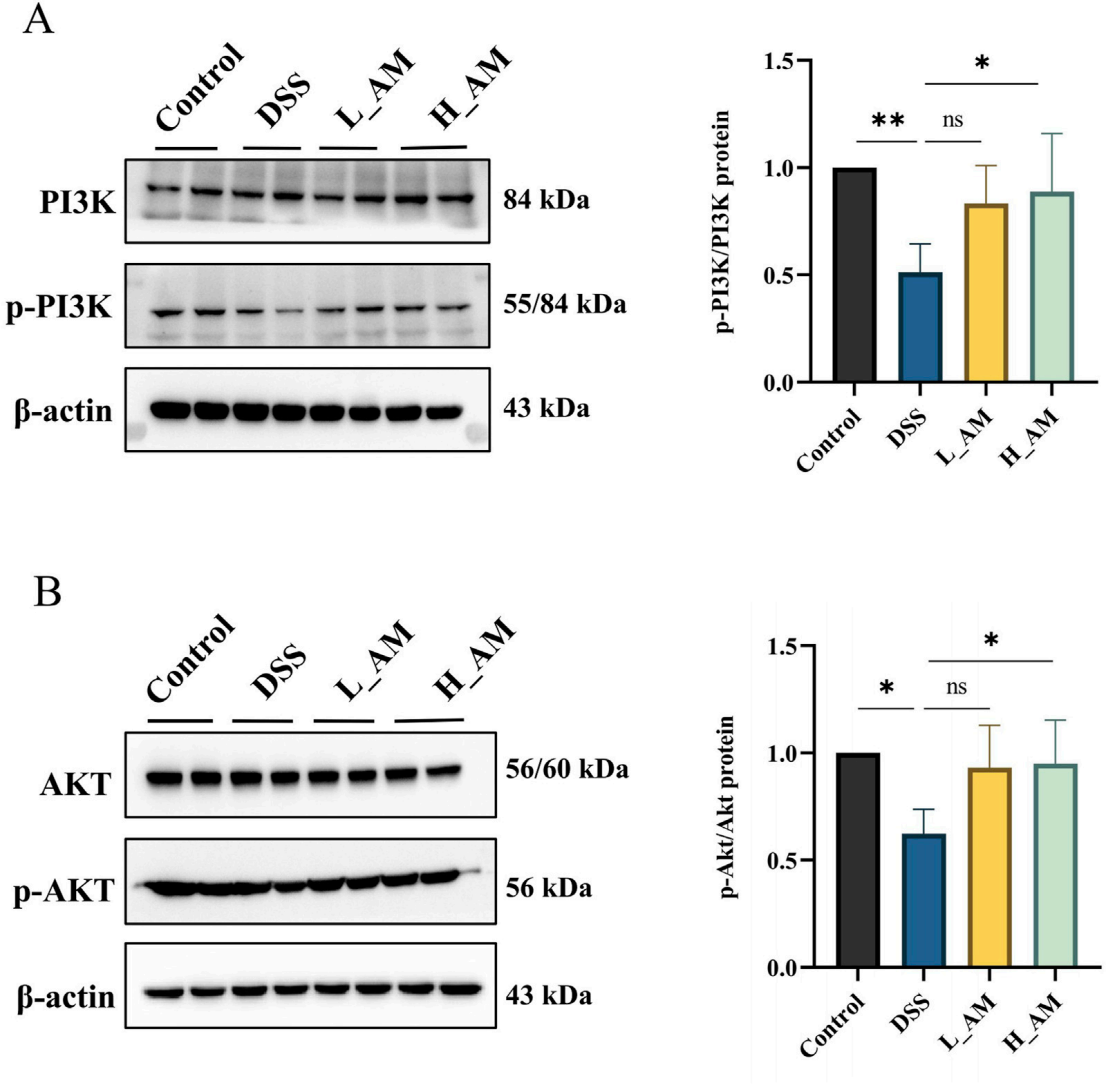
AM extract modulates the PI3K/AKT signaling pathway in UC mice. **(A)** Protein expression levels of p-PI3K, PI3K. **(B)** p-AKT, AKT. The data are expressed as mean ± SEM. (n = 4 in **(A,B)**. *P < 0.05, **P < 0.01.

Network pharmacology findings indicated that AM extract likely exerts its therapeutic effects on UC mice via the PI3K/AKT signaling pathway. The experimental results (Figures 12A,B) demonstrated that, compared with the control group, the DSS group presented significantly lower p-PI3K/PI3K and p-AKT/AKT ratios (*P* < 0.05 or *P* < 0.01), reflecting impaired activation of the pathway. Treatment with AM extract significantly increased these phosphorylation levels (*P* < 0.05), indicating that the protective effects of AM extract in UC are mediated through activation of the PI3K/AKT signaling pathway. These findings suggest that effective doses of AM extract may alleviate UC injury by enhancing downstream signaling through this pathway.

## 4 Discussion

UC originates in the rectum and progressively extends proximally, resulting in gastrointestinal tract damage that can manifest as stenosis, abscesses, or fistulas (Khor et al., 2011). TCM offers a multifaceted approach to UC management by regulating immune responses, suppressing excessive inflammation, restoring intestinal barrier integrity, and enhancing self-repair mechanisms, ultimately curbing disease progression (Yuan et al., 2022). Astragaloside IV has been shown to alleviate inflammation and ameliorate colitis symptoms by modulating the PI3K/AKT pathway and improving gut microbiota imbalances (Zhang et al., 2024). Similarly, AM extract has anti-inflammatory effects by mitigating LPS- and IFN-γ-induced inflammation and reducing TNF-*α* release through activation of the NF-κB and Nrf2 pathways (Adesso et al., 2018). These findings highlight the therapeutic potential of AM extract and its active compounds in regulating inflammatory pathways, which warrants further exploration.

This study revealed that AM extract reduced DAI scores and improved pathological injury, indicating the potential therapeutic effect of AM extract on UC injury. Moreover, an imbalance in the gut microbiota is widely recognized as a critical factor in the pathogenesis of UC (Foppa et al., 2024). The gut microbiota, often described as a ‘microbial organ,’ facilitates the breakdown of indigestible dietary fiber and the production of beneficial metabolites (Cai et al., 2022). In the present study, the *α* diversity indices indicated that AM extract restored microbial diversity in UC model mice. Furthermore, the AM extract was found to increase the relative abundance of *Lactobacillus* and Akkermansiaceae in the serum of UC mice. Notably, Akkermansiaceae and *Lactobacillus* are known to induce intestinal adaptive immune responses and possess the ability to produce SCFAs (Li et al., 2024; Huynh and Zastrow, 2023). A previous study confirmed that variations in SCFAs levels contribute to the maintenance of mucosal homeostasis (Tan et al., 2023). Among these, butyric acid plays a pivotal role by promoting epithelial cell proliferation and differentiation, as well as by upregulating the expression of tight junction proteins, thereby preserving the integrity of the intestinal barrier (Shin et al., 2023). Our findings revealed that AM extract effectively modulates SCFAs metabolism by increasing the levels of beneficial SCFAs (acetate, propionate, and butyrate) and reducing the levels of harmful metabolites such as isovaleric acid, which likely contributes to protection of the intestinal mucosal barrier in UC mice.

Moreover, BAs are strongly associated with UC. Primary BAs are synthesized in the liver and subsequently metabolized into secondary BAs in the intestine (Jia et al., 2018). Consequently, an imbalance in the gut microbiota may disrupt BAs metabolism, potentially resulting in elevated levels of secondary BAs and contributing to the development of UC (Do et al., 2023). Here, the ratio of primary BAs to secondary BAs increased in the DSS group, suggesting that a gut microbiota imbalance during colitis impairs metabolic function, reducing the conversion of primary BAs to secondary BAs (Ma et al., 2025). However, this ratio tended to decrease after AM extract treatment. These results further revealed the positive regulatory effects of AM extract on microbiota, SCFAs, and BAs metabolism in UC mice.

LPS and D-lac are key biomarkers of intestinal permeability (Mao et al., 2024), whereas tight junction proteins such as ZO-1, occludin, and claudin serve as important indicators of intestinal barrier integrity (Mo et al., 2022). Once the intestinal barrier is compromised, a cascade of inflammation is triggered. NF-κB, as a nuclear transcription factor, plays an important role in the inflammatory signaling pathway, regulating cytokine synthesis and controlling the expression of various inflammatory genes (Liu et al., 2025), whereas Nrf2 is an important transcription regulator involved in redox homeostasis and plays an irreplaceable role in promoting antioxidant responses in organisms (Huang et al., 2025). Subsequent validation demonstrated that the AM extract effectively repaired intestinal barrier damage and inhibited the release of inflammatory markers. In summary, AM extract effectively restores intestinal barrier integrity and suppresses inflammation by regulating key biomarkers of permeability and modulating inflammatory and antioxidant signaling pathways.

TCM is characterized by its multicomponent and multitarget regulatory effects in the treatment of various diseases (Guo et al., 2022), and Jin et al. (2022) noted that the original blood components and metabolites of TCM may play crucial roles in mediating its pharmacological effects. Here, we further examined the blood components of AM extract, identifying compounds such as calycosin, formononetin, and kaempferol. These compounds were found to be converted into their respective glycosides: calycosin-7-O-glucoside, formononetin-7-glucoside, and kaempferol 3-O-alpha-L-hmnpyranoside. Among these compounds, calycosin-7-O-glucan has been demonstrated to mitigate cell damage through the regulation of oxidative stress and apoptosis (He et al., 2021). Calycosin-7-O-glucoside functions as a natural anti-inflammatory agent by inhibiting the release of inflammatory factors via modulation of the NF-κB pathway (Danciu et al., 2018). Additionally, prototype components such as quercetin and isorhamnetin were identified. Quercetin inhibits NF-κB phosphorylation, thereby decreasing its activity and reducing the expression of inflammatory factors (Zhang et al., 2022). Isorhamnetin also has both anti-inflammatory and antioxidant properties (Wang et al., 2021). Therefore, we speculate that the conversion and absorption of these components and their metabolites may be the fundamental basis for the protective effect of AM extract on UC.

Given the multicomponent and multitarget nature of TCM, interactions among various constituents are likely. Therefore, it is essential to comprehensively investigate the complex network relationships among “components-target-pathway.” Network pharmacology, which is based on systems biology theory, incorporates methodologies from related fields such as computational biology and experimental validation (Li et al., 2023). In this study, network pharmacology was utilized to predict potential targets and signaling pathways of AM extract in the treatment of UC. The results confirmed that the AM extract interacts with multiple targets, including IL1B, IL6, and TP53, to exert protective effects. Additionally, the associated signaling pathways appeared to be interconnected through these target proteins, collectively contributing to UC treatment. KEGG analysis indicated that the PI3K/AKT pathway is closely linked to the therapeutic effects of AM. As a key regulator of autophagy, the inhibition of the PI3K/AKT pathway promotes autophagy activation, alleviating inflammation through intracellular pathogen clearance (Wang et al., 2018). To validate the “component-disease-target” approach in network pharmacology, WB experiments were performed, which revealed that the AM extract effectively increased the p-PI3K/PI3K and p-AKT/AKT ratios. These findings suggest that AM extract may confer protective effects against UC by increasing the phosphorylation levels of the PI3K/AKT signaling pathway. However, this study has certain limitations, and future research will focus more on functional experiments on how AM extract regulates the PI3K/AKT signaling pathway to alleviate UC damage.

## 5 Conclusion

AM extract has the potential to alleviate symptoms associated with UC in mice, likely through the modulation of the gut microbiota balance, SCFAs and BAs metabolism, and the upregulation of the PI3K/AKT pathway.

## Data Availability

The original contributions presented in the study are included in the article/Supplementary Material, further inquiries can be directed to the corresponding authors.

## Glossary

AKT: Protein kinase B
AM: Astragalus membranaceus
ANOVA: analysis of variance
ASVs: Amplicon Sequence Variants
BAs: Bile acids
BCA: Bicinchoninic acid
BP: biological processes
CC: Cellular components
DAI: Disease activity index
DSS: Dextran sodium sulfate
ECL: Enhanced chemiluminescence
ELISA: Enzyme-linked Immunosorbent Assay
FXR: Farnesoid X Receptor
GO: Gene Ontology
H&E: Hematoxylin-eosin
IBD: Inflammatory bowel disease
IF: Immunofluorescence
IS: Internal standard
KEGG: Kyoto Encyclopedia of Genes and Genomes
MF: Molecular functions
NS: Normal saline
N2: Nitrogen
p-AKT: Phosphorylated AKT
PCA: Principal component analysis
PDK1: Phosphoinositide-dependent kinase 1
PI3K: Phosphatidylinositol 3-kinase
PIP2: Phosphatidylinositol 4,5-bisphosphate
PIP3: Phosphatidylinositol 3,4,5-trisphosphate
PPI: Protein-protein interaction
PVDF: Polyvinylidene fluoride
SCFAs: Short-chain fatty acids
TCM: Traditional Chinese medicine
TCMSP: Traditional Chinese Medicine System Pharmacology
TRG5: Takeda G protein-coupled receptor 5
UC: Ulcerative colitis
UPLC/Q-TOF-MS: Ultra Performance Liquid Chromatography-Quadrupole-Time-of-Flight Mass Spectrometry.
