# Supplementary figures and images for: Astragalus membranaceus extract attenuates ulcerative colitis by integrating multiomics and the PI3K/AKT signaling pathway
Source: Front Pharmacol. 2025 Jun 9;16:1585748. doi: 10.3389/fphar.2025.1585748 (PMC12183238; doi:10.3389/fphar.2025.1585748)

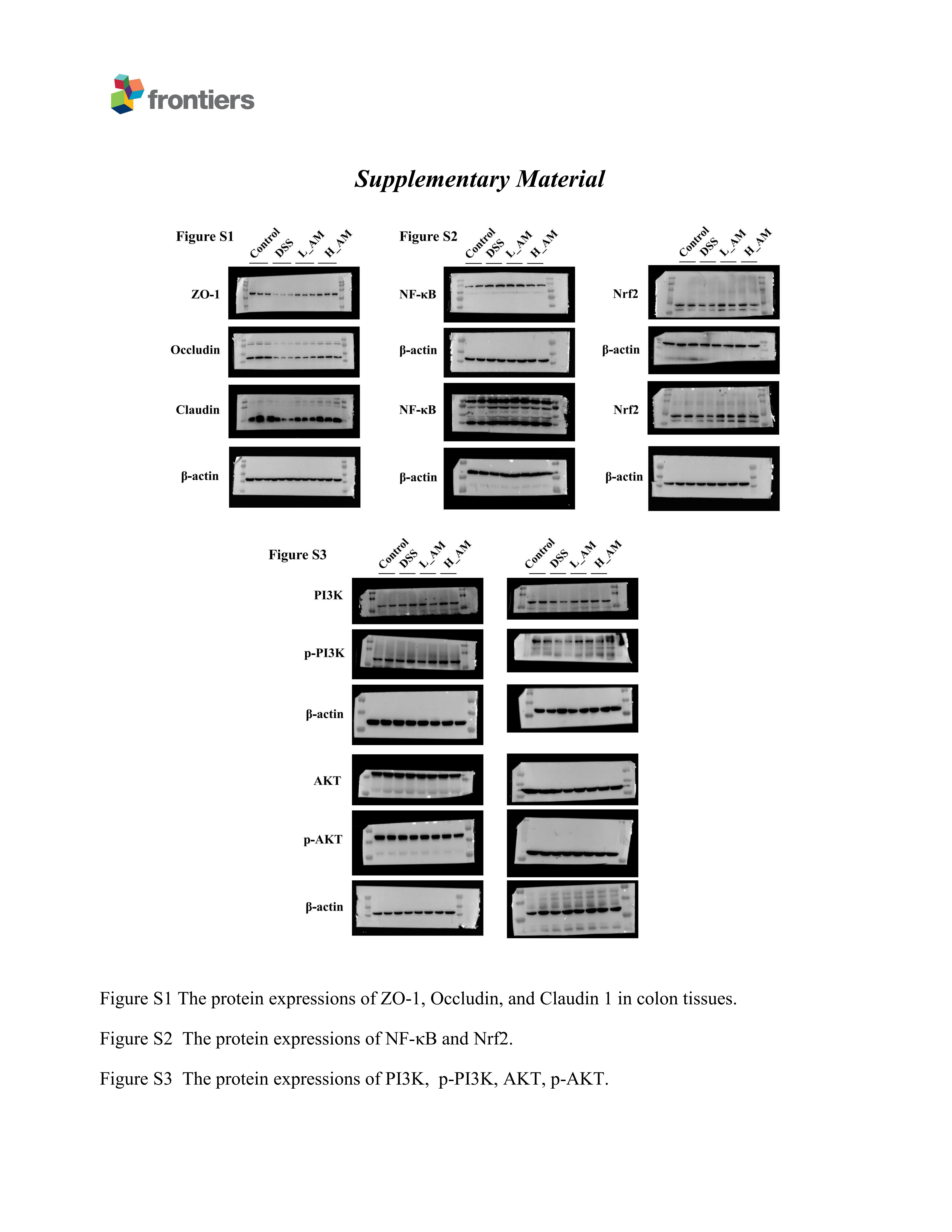

Supplement: Supplementary file 1 [file Image1.jpeg]
